# Baseline Motivation Type as a Predictor of Dropout in a Healthy Eating Text Messaging Program

**DOI:** 10.2196/mhealth.5992

**Published:** 2016-09-29

**Authors:** Kisha Coa, Heather Patrick

**Affiliations:** ^1^ICF InternationalRockville, MDUnited States; ^2^Applied Behavior Change ScienceScience and ProductEnvolve PeopleCareBethesda, MDUnited States

**Keywords:** mHealth, behavior change, diet, engagement, motivation, Self-Determination Theory

## Abstract

**Background:**

Growing evidence suggests that text messaging programs are effective in facilitating health behavior change. However, high dropout rates limit the potential effectiveness of these programs.

**Objective:**

This paper describes patterns of early dropout in the HealthyYou text (HYTxt) program, with a focus on the impact of baseline motivation quality on dropout, as characterized by Self-Determination Theory (SDT).

**Methods:**

This analysis included 193 users of HYTxt, a diet and physical activity text messaging intervention developed by the US National Cancer Institute. Descriptive statistics were computed, and logistic regression models were run to examine the association between baseline motivation type and early program dropout.

**Results:**

Overall, 43.0% (83/193) of users dropped out of the program; of these, 65.1% (54/83; 28.0% of all users) did so within the first 2 weeks. Users with higher autonomous motivation had significantly lower odds of dropping out within the first 2 weeks. A one unit increase in autonomous motivation was associated with lower odds (odds ratio 0.44, 95% CI 0.24–0.81) of early dropout, which persisted after adjusting for level of controlled motivation.

**Conclusions:**

Applying SDT-based strategies to enhance autonomous motivation might reduce early dropout rates, which can improve program exposure and effectiveness.

## Introduction

The proliferation of mobile phone ownership in the United States and worldwide has provided a unique opportunity to reach broad audiences with health behavior messages and interventions [1-3]. Growing evidence suggests that text messaging programs are effective in facilitating health behavior change in multiple domains, including diet and physical activity [1,4,5]. Some of the advantages of text messaging programs over traditional health behavior change programs include their potential reach, particularly among hard-to-reach populations, and cost-effectiveness [6]. However, high user dropout rates are commonly noted in text-based programs [4]. This issue represents a significant challenge, as the majority of disenrollment occurs early in these interventions, limiting the potential effectiveness of text messaging programs [7]. In order for programs to be maximally effective, there is a need to identify modifiable predictors of engagement that can serve as the basis for strategies to reduce dropout.

Motivation is one factor that has been associated with engagement in health behavior change programs and longer-term pursuits of health behavior change [8]. Traditional theories of motivation have typically been of limited value, in part because they have conceptualized motivation as a relatively static variable measured in terms of quantity, rather than quality [9]. Self-Determination Theory (SDT) offers an alternative approach to characterizing motivation, and may prove to be more useful in understanding and enhancing motivation for sustained behavioral change [10-12]. SDT may also provide key insights into engagement and persistence with mobile technology-based interventions. Rather than focusing solely on motivational quantity, SDT emphasizes the importance of motivational quality in engagement with, and maintenance of, behavior [10,11]. SDT distinguishes between autonomous motivation, which arises from within the person and is congruent with other goals and values, and controlled motivation, which emerges from external and/or intrapsychic pressures (eg, incentives, approval from others, feelings of guilt or shame). Autonomous motivation has been associated with improved physical and mental health outcomes, and initiation and maintenance of health behaviors [13-15]. This study describes patterns of early dropout in the HealthyYou text (HYTxt) messaging program and examines them through the lens of SDT.

## Methods

### Intervention Description

HYTxt is a free text message-based resource that provides behavioral interventions to promote healthy diet and physical activity. HYTxt was developed as a component of the US National Cancer Institute’s Smokefree.gov Initiative (SFGI). SFGI is a large, national, multi-platform program that includes websites, text message programs, smartphone apps, and social media platforms that engage with 3-6 million users annually. HYTxt is a 6-week intervention program that delivers 1-4 daily texts, including standardized behavioral intervention and social support messages that target specific health behavior goals each week, via unidirectional and bidirectional messages. At the time of enrollment, users answer a series of basic demographic questions and complete a brief motivational assessment. Users then select a behavior change goal related to healthy eating or physical activity. Throughout the program, users receive text messages that provide SDT-based intervention content, and assess their status in achieving health behavior goals. Users may discontinue the program at any time by texting the word *stop* to the program.

### Sample

As a part of ongoing program quality assurance and improvement efforts, the sample for this project consisted of 193 consecutive individuals who enrolled in the HYtxt healthy eating stream of the text messaging program between June 2014 and December 2014, and met our inclusion criteria. Given our interest in the impact of motivation type on early dropout, we restricted our analysis to individuals with complete data for the motivation questions (described below). We did not collect information on the gender of our participants, but the program was promoted on the Smokefree Women Website [16] and Smokefree Women Facebook page, so the sample is presumed to be primarily women.

### Measures

The dependent variable was early dropout, which was defined as actively dropping out within 2 weeks of program enrollment. Our primary independent variable was motivation quality. Table 1 includes the items used to assess motivational quality, adapted from the Self-Regulation Questionnaire [17]. Response options were *very true* (4), *a little true* (3), *a little untrue* (2), and *very untrue* (1). The *autonomous motivation* subscale was comprised of the mean of the identified and integrated questions; the *controlled motivation* subscale was comprised of the mean of the introjected and external questions. Higher values signified higher levels of motivational quality.

Users self-reported their age, educational level, and race/ethnicity during program enrollment. Users could enroll in the HYTxt program by texting a keyword on their phone or by completing the Web enrollment form. Only individuals who enrolled using the Web enrollment were asked demographic questions; therefore, demographic data were only available for that subset of users.

### Statistical Analyses

All analyses were conducted in SAS 9.3 (SAS Institute, Inc). Descriptive statistics were calculated and chi-square tests were conducted to examine associations between demographic characteristics and early dropout status. Logistic regression was used to test the association between early dropout and motivation type. Separate unadjusted logistic regression models for controlled motivation on dropout, and autonomous motivation on dropout, were computed. Subsequently, a model including both controlled motivation and autonomous motivation was run. For the subset of individuals with complete demographic data (n=126) we also examined the association between autonomous motivation and early dropout, controlling for demographic characteristics (ie, education level, race, and age).

**Table 1:** Question wording for each motivation type assessed at baseline.

Motivation type	Question wording: I would try to eat more fruits, vegetables, and whole grains because…
Autonomous (identified)	Eating fruits, vegetables, and whole grains helps me feel better
Autonomous (integrated)	Eating more fruits, vegetables, and whole grains is an important thing for me to do
Controlled (introjected)	I would feel bad about myself if I didn’t
Controlled (external)	Others want me to eat more fruits, vegetables and whole grains

## Results

Most users had at least some college education, were predominately white, and had an average age of 34.6 years (Table 2). There were no significant demographic differences between those who dropped out within the first 2 weeks and those who did not. Overall, 28.0% (54/193) of users dropped out of the program within the first 2 weeks.

Of those who dropped out before completing the program (83/193, 43.0%), 65.1% (54/83) of users did so within the first 2 weeks.

At the beginning of the program, most users endorsed both autonomous and controlled motivations for healthy eating; however, on average users reported higher levels of autonomous motivation (mean 3.64, standard deviation 0.49) than controlled motivation (mean 2.42, standard deviation 0.88). Users with higher autonomous motivation had significantly lower odds of dropping out of the program within the first 2 weeks. A one unit increase in autonomous motivation was associated with a significantly lower odds of early dropout (odds ratio [OR] 0.44, 95% CI 0.24–0.81; *P*=.008). This association persisted after adjusting for level of controlled motivation (OR 0.48, 95% CI 0.26–0.90; *P*=.022). Controlled motivation was not associated with early dropout (OR 0.71, 95% CI 0.49–1.02; *P*=.064). Given the association between autonomous motivation and early dropout, we further explored whether this association held true after controlling for demographic characteristics (67/193, 34.7% of cases were missing data, and were excluded), and autonomous motivation remained significantly associated with early dropout (OR 0.44, 95% CI 0.20–0.95; *P*=.035).

**Table 2:** Sample demographic characteristics.

Variable		% or mean (n)	Dropped out within the first 2 weeks, % (n)		*P* value
			Yes	No	
**Total, % (n)**					
		100.0 (193)	28.0 (54)	72.0 (139)		
**Education, % (n)**	
	High school or less	18.1 (35)	22.2 (12)	16.6 (23)	0.77	
	Some college	32.6 (63)	33.3 (18)	32.4 (45)		
	College graduate or higher	19.2 (37)	18.5 (10)	19.4 (27)		
	Missing	30.1 (58)	25.9 (14)	31.7 (44)		
**Race, % (n)**	
	White	48.7 (94)	59.3 (32)	44.6 (62)	0.19	
	Non-white	18.1 (35)	14.8 (8)	19.4 (27)		
	Missing	33.2 (64)	25.9 (14)	36.0 (50)		
**Age in years, mean (range)**		34.6 (13-62)	32.6 (16-62)	35.5 (13-62)	0.22	

## Discussion

### Principal Results

In this study, approximately two-thirds (65.1%, 54/83) of users who dropped out did so within the first 2 weeks. Users with higher levels of autonomous motivation at baseline were less likely to drop out early, even after adjusting for controlled motivation. This result is consistent with previous research that has demonstrated that autonomous motivation is predictive of initiating and maintaining health behaviors [14,18].

### Limitations

Participants in this study were users of the HYTxt text messaging program, and were not recruited for a particular study. Although this sample represents a self-selected group, they are likely more reflective of individuals who would use the publicly available program in general than participants of a tightly controlled randomized trial would be. Not all users completed the motivation questions, resulting in a great deal of missing data for demographic correlates; therefore, our results may not be generalizable to all users of HYTxt. This analysis focused solely on motivation related to healthy eating, and other factors (eg, comfort with mobile technology) might influence dropout rates. Furthermore, we focused on those who actively opted out of the program, and cannot comment on those who may have continued to receive messages but disengaged from the program.

### Comparison with Prior Work

Dropout is a significant problem in behavior change text messaging interventions. This study provides insight into a modifiable factor that influences likelihood of dropping out: autonomous motivation. The extant literature on SDT-based interventions offers potential insights into techniques that could be leveraged to promote autonomous motivation early in text messaging behavior change programs to decrease early dropout. Candidate SDT techniques include: supporting users in aligning behavior change goals with broader life goals and values; providing opportunities for choice, including opportunities to disengage from the current program and either (1) return at a later time or (2) change to another health behavior that better fits the user’s current interests and life situation; and providing content that normalizes the trial-and-error nature of the health behavior change processes, while offering strategies for dealing with failures and setbacks [18-20]. While these techniques have been well-validated in more traditional intervention delivery modalities (eg, in-person or telephone-based), there is a need to translate these techniques into strategies that would be applicable when using a text messaging platform. For example, more messaging can be added at early stages that encourage users to consider how this behavior change aligns with their broader life goals and values, and ensure that users are selecting behavior change goals that are consistent with their core values. In addition, users’ progress towards meeting their goals can be assessed at regular intervals, and individuals who are struggling can be provided with additional content that both normalizes setbacks and provides strategies for dealing with these setbacks.

### Conclusions

Text messaging interventions have the potential to reach large numbers of individuals, especially traditionally hard-to-reach populations. However, high early dropout rates inhibit the potential effectiveness of such programs. This study demonstrated that those who had higher levels of autonomous motivation were less likely to drop out in the first 2 weeks of the program, during which dropout rates were highest. Applying SDT-based strategies early in text messaging programs (to enhance autonomous motivation) might reduce early dropout rates, which in turn can improve program exposure and effectiveness.

## Abbreviations

HYTxt: HealthyYou text
OR: odds ratio
SDT: Self-Determination Theory
SFGI: Smokefree.gov Initiative
